# Neutrophil gelatinase-associated lipocalin is elevated in children with acute kidney injury and sickle cell anemia, and predicts mortality

**DOI:** 10.1016/j.kint.2022.05.020

**Published:** 2022-06-17

**Authors:** Anthony Batte, Sahit Menon, John M. Ssenkusu, Sarah Kiguli, Robert Kalyesubula, Joseph Lubega, Zachary Berrens, Edrisa Ibrahim Mutebi, Rodney Ogwang, Robert O. Opoka, Chandy C. John, Andrea L. Conroy

**Affiliations:** 1 Child Health and Development Centre, Makerere University College of Health Sciences, Kampala, Uganda; 2 San Diego School of Medicine, University of California, San Diego, California, USA; 3 Department of Epidemiology and Biostatistics, Makerere University School of Public Health, Kampala, Uganda; 4 Department of Paediatrics and Child Health, Makerere University College of Health Sciences, Kampala, Uganda; 5 Department of Medicine, Makerere University College of Health Sciences, Kampala, Uganda; 6 Division of Pediatric Hematology and Oncology, Baylor College of Medicine, Houston, Texas, USA; 7 Department of Pediatrics, Pediatric Critical Care Medicine, Indiana University School of Medicine, Indianapolis, Indiana, USA; 8 Kenyan Medical Research Institute (KEMRI)-Wellcome Trust Research Programme, Centre for Geographic Medicine Coast, Kilifi, Kenya; 9 Department of Pediatrics, Ryan White Center for Pediatric Infectious Disease and Global Health, Indiana University School of Medicine, Indianapolis, Indiana, USA

**Keywords:** acute kidney injury, biomarker, chronic kidney disease, neutrophil gelatinase-associated lipocalin, sickle cell anemia, sub-Saharan Africa

## Abstract

Urine neutrophil gelatinase-associated lipocalin (NGAL) is a biomarker of acute kidney injury that has been adapted to a urine dipstick test. However, there is limited data on its use in low-and-middle-income countries where diagnosis of acute kidney injury remains a challenge. To study this, we prospectively enrolled 250 children with sickle cell anemia aged two to 18 years encompassing 185 children hospitalized with a vaso-occlusive pain crisis and a reference group of 65 children attending the sickle cell clinic for routine care follow up. Kidney injury was defined using serial creatinine measures and a modified-Kidney Disease Improving Global Outcome definition for sickle cell anemia. Urine NGAL was measured using the NGAL dipstick and a laboratory reference. The mean age of children enrolled was 8.9 years and 42.8% were female. Among hospitalized children, 36.2% had kidney injury and 3.2% died. Measured urine NGAL levels by the dipstick were strongly correlated with the standard enzyme-linked immunosorbent assay for urine NGAL (hospitalized children, 0.71; routine care reference, 0.88). NGAL levels were elevated in kidney injury and significantly increased across injury stages. Hospitalized children with a high-risk dipstick test (300ng/mL and more) had a 2.47-fold relative risk of kidney injury (95% confidence interval 1.68 to 3.61) and 7.28 increased risk of death (95% confidence interval 1.10 to 26.81) adjusting for age and sex. Thus, urine NGAL levels were found to be significantly elevated in children with sickle cell anemia and acute kidney injury and may predict mortality.

Sickle cell anemia (SCA) is an inherited hemoglobinopathy of increasing global incidence that results from a single amino acid substitution in the gene encoding b-hemoglobin. By 2050, it is projected that 400,000 newborns will be born with SCA annually,^1^ with ≈85% of cases occurring in sub-Saharan Africa.^2^ SCA is a leading cause of death in children aged <5 years in sub-Saharan Africa^3^ and an established risk factor for kidney disease.^4–10^ Polymerization of deoxygenated sickle hemoglobin results in decreased deformability of red blood cells and leads to vaso-occlusive crises, one of the most common complications in children living with SCA.^11,12^ Several studies have reported an increased risk of acute kidney injury (AKI) in children with SCA during a vaso-occlusive crisis.^13–16^ AKI in the context of SCA is associated with increased resource utilization and mortality.^15,17^

AKI is an abrupt decrease in kidney function based on an increase in serum creatinine or a decrease in urine output. However, creatinine is a late marker of AKI, with limited sensitivity to detect smaller changes in kidney function,^18,19^ and is affected by nonkidney factors, including malnutrition.^20^ In SCA, the presence of glomerular hyperfiltration and increased tubular reabsorption of creatinine further complicate AKI recognition.^21^ There is a critical need to evaluate alternative biomarkers to improve early detection of AKI that are accessible across a variety of clinical settings.^22^ The development of point-of-care tests to diagnose AKI will facilitate equitable access by reducing reliance on centralized diagnostics and allowing near-patient early AKI recognition.

Neutrophil gelatinase-associated lipocalin (NGAL) is a 25-kilodalton secreted innate immune protein expressed by neutrophils.^23^ NGAL is rapidly upregulated by the kidney in the context of ischemia and tubular injury.^24,25^ Meta-analyses report excellent diagnostic utility in sepsis-associated AKI, with areas under the receiver operating characteristic curve >0.90.^26,27^ NGAL can also predict the need for kidney support therapy and identifies patients at highest risk of mortality.^26,27^ Urine NGAL (uNGAL) has been adapted to a dipstick test facilitating semiquantitative point-of-care evaluation of NGAL and has been validated in the context of trauma-associated AKI.^28^

n the present study, we hypothesized that uNGAL would be elevated in AKI and associated with increased risk of mortality. We evaluated this in a prospective cohort of children with SCA hospitalized with a vaso-occlusive crisis alongside a reference group of children with SCA in steady state. uNGAL was measured on enrollment using a point-of-care dipstick test, and the results were validated using a quantitative laboratory assay. Among hospitalized children, we evaluated the relationship between uNGAL and clinical features of disease severity, AKI, and mortality.

## Methods

### Study population

Between January and August in 2019, 250 children with SCA were enrolled in the study at Mulago National Referral and Teaching Hospital in central Uganda. Study participants included 185 consecutively recruited children with SCA hospitalized for a vasoocclusive pain crisis, as previously described.^17^ AKI was assessed using the Kidney Disease: Improving Global Outcomes (KDIGO) criteria using serial creatinine measures.^29^ Inclusion criteria were documented SCA by hemoglobin electrophoresis, age of 2 to 18 years, pain score ≥2 on an age-specific pain scale, and a willingness to complete the study procedures. Pain in children aged 2 to 3 years was assessed using the face, legs, activity, cry, and consolability scale.^30^ Participants aged >3 to 7 years were assessed using the Wong-Baker Faces pain scale, and children aged ≥8 years were assessed using the numeric pain scale.^31,32^ In addition to the hospitalized children, 65 age-matched children with SCA in steady state who were attending the sickle cell clinic for routine follow-up were enrolled as a reference group. Exclusion criteria for the reference group included an active illness or the presence of pain.

Mulago National Referral Hospital is located in Kampala in central Uganda and has a high outpatient and inpatient burden, with an average of 3 hospital admissions daily in children with SCA for vaso-occlusive crises. Most children admitted with vaso-occlusive crises are referred from the hospital’s dedicated sickle cell clinic, which provides medical care to ≈1400 children per year. Routine care includes daily folic acid and penicillin V for children aged <5 years and malaria prophylaxis with sulfadoxine-pyrimethamine. The use of sickle cell–modifying therapy hydroxyurea is available in a limited capacity for children meeting clinical indications. There are no standard protocols for identifying, monitoring, and managing AKI in the acute care unit. The decision to assess creatinine is based on each patient’s clinical risk, according to the managing physician. Routine monitoring of urine output or daily weights is not performed.

### Study procedures

On enrollment, all children had a complete history and physical examination conducted by a study medical officer to assess medication use, signs of infection, and the site and severity of pain. Blood pressure was calculated as the mean of 3 independent measurements, and hypertension was defined as a systolic blood pressure >95th percentile or a diastolic blood pressure >95th percentile from 3 independent measurements for children aged <13 years or a systolic blood pressure ≥130 mm Hg or a diastolic blood pressure ≥80 mm Hg for children aged ≥13 years.^33^ Children were weighed on a standardized electronic scale, and height was measured using a stadiometer. Heights and weights were converted into z-scores (height for age, weight for age, weight for height, or body mass index for age) based on WHO growth references.^34,35^

All children had blood collected at enrollment for malaria evaluation, a complete blood cell count, and a point-of-care i-STAT test using the CHEM8+ cartridge that measures metabolic status and renal function (Abbott Point of Care Inc.). A spot urine sample was collected using a urine bag or urine container for older children and sent to the laboratory within 2 hours of collection for urinalysis and uNGAL dipstick. Urine samples were spun at room temperature for 5 minutes at 400*g* and collected and stored at -80 °C until testing.

### Assessment of infection

On admission, all children were assessed for signs of infection. Acute infection was defined as the presence of sepsis, malaria, or urinary tract infection. Sepsis was defined using the Systemic Inflammatory Response Syndrome (SIRS)/sepsis in international pediatric sepsis consensus guidelines, as previously described.^36^ The diagnosis of a urinary tract infection was based on a positive nitrite or leukocyte test result by urinalysis in children who presented with fever. Malaria was diagnosed by Giemsa-stained thick and thin blood smears, according to standard protocols. Blood and urine cultures were not available.

### Assessment of kidney function

On the basis of the KDIGO guidelines, AKI was defined as an increase in serum creatinine ≥0.3 mg/dl within 48 hours or a 50% increase in baseline creatinine within 7 days.^29^ The definition was modified to exclude children with a 1.5-fold increase in creatinine from 0.2 to 0.3 mg/dl, as previously described.^17^ Daily 24-hour urine output was not quantified during hospitalization. Kidney function was assessed on enrollment, at 48 hours, and at day 7 or discharge (whichever happened earlier) by iSTAT using an enzymatic assay traceable to the US National Institute of Standards and Technology standard reference material SRM909, with a reportable range of 0.20 to 20.0 mg/dl. Creatinine values below the reportable range were assigned a value of 0.19 mg/dl.

The participants’ lowest measured creatinine was taken as the baseline. In instances where only a single creatinine measure was available (n = 7), the Pottel age-based glomerular filtration rate (GFR) estimating equation^37^ was used to back-calculate baseline creatinine, assuming a normal GFR of 120 ml/min per 1.73 m^2^.^38^ Using the KDIGO guidelines, AKI was staged on the basis of creatinine fold change from baseline to the highest value recorded, where stage 1 included a 1.5- to <2-fold change in creatinine from baseline or a ≥0.3-mg/dl increase in creatinine within 48 hours; stage 2, a 2- to <3-fold change in creatinine from baseline; stage 3, a ≥3-fold change in creatinine from baseline, an increase in creatinine to ≥4.0 mg/dl, or an estimated GFR ≤35 ml/min per 1.73 m^2^. In addition, serum cystatin C was assessed as an alternative measure of GFR by enzyme-linked immunosorbent assay using a Quantikine assay by R&D Systems.

As SCA is associated with increased tubular secretion of creatinine and can affect reliable estimated GFR estimates,^21^ estimated GFR on admission was calculated using the creatinine and cystatin C–based formula: estimated GFR = 39.8^⋆^ [(height/creatinine)^0.456^] ^⋆^ [(1.8/cystatin C)^0.418^] ^⋆^ [(30/blood urea nitrogen)^0.079^] ^⋆^ (1.076^male^) ^⋆^ [(height/1.4)^0.179^].^39^

### NGAL measurements

NGAL was measured on fresh urine samples using the uNGAL dipstick test kit from BioPorto Diagnostics Inc., according to the instructions.^28^ The test is an antibody sandwich lateral flow dipstick test, where the intensity of the color in the test line correlates with the concentration of NGAL in the sample. Briefly, the uNGAL dipstick reagent tube was brought to room temperature, 3 drops of sample dilution buffer were added to the reagent tube provided, and 10 μl of urine was added to the reagent tube using a volumetric pipette, mixed, and incubated at room temperature for 5 minutes. After 5 minutes, the lateral flow test strip was placed with arrows pointing down into the sample and incubated for 10 minutes, ensuring the control line was visible before reading the semi-quantitative results using the quantification guide. Reference levels of uNGAL were tested in batches on stored samples by enzyme-linked immunosorbent assay, according to the manufacturer’s protocol (Kit 036; BioPorto Diagnostics Inc.). Urine samples were diluted 1:1000, and the upper and lower limits of the assay were 2000 and 5 ng/ml, respectively. All testing was conducted by technicians blinded to participant details.

### Statistical analysis

Data were double entered into REDCap electronic data capture tools hosted at Indiana University. Data were analyzed using STATA v14.0 (StataCorp) and GraphPad Prism v7.03. Data are presented descriptively using mean and SD or median and interquartile range (IQR) for continuous variables and number and frequency for discrete variables. The frequency of missing data is presented in the article. The relationship between NGAL levels and dichotomous outcomes was assessed using a Wilcoxon rank-sum test, a Student t test, or a nonparametric test of trend across stages of AKI using the method of Cuzick.^40^ To evaluate the discriminatory ability of the biomarker tests, we generated nonparametric receiver operating characteristic curves and reported the area under the curve. To evaluate the relationship between NGAL and AKI status, hematuria, mortality, and other clinical variables among the cases, we fitted a modified Poisson model with robust standard errors (SEs).^41,42^ This model was preferred to prevent overestimation of SEs often observed when logistic regression models are fitted for common outcomes.

### Ethics

Written informed consent was obtained from the parents or legal guardians of all study participants, and assent was obtained for children aged ≥8 years. The Institutional Review Board from Makerere University School of Biomedical Sciences Research and Ethics Committee granted ethical approval (first approval date, May 13, 2018; Institutional Review Board number SBS-S46). The Uganda National Council for Science and Technology provided regulatory approval for the study (approval date, September 7, 2018; approval number HS 2443).

## Results

### Description of the study population

**Figure 1:**
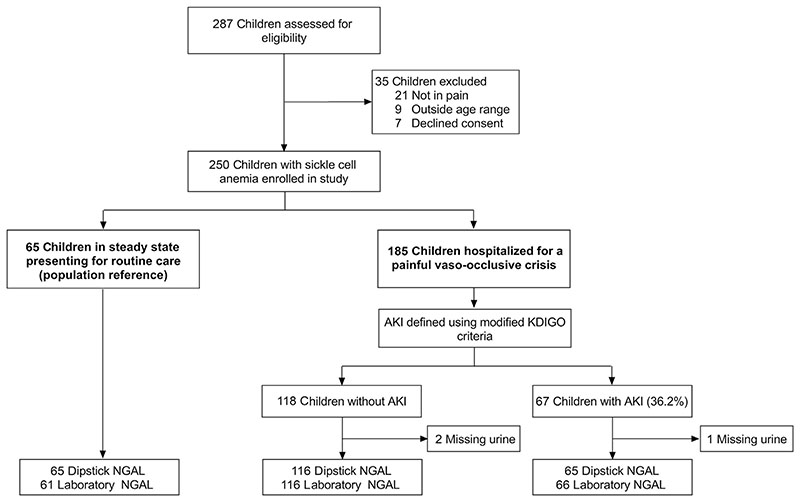
Flowchart of study population. A flowchart showing the number of children with neutrophil gelatinase-associated lipocalin (NGAL) assessed on the basis of acute kidney injury (AKI) status. KDIGO, Kidney Disease: Improving Global Outcomes.

We enrolled 250 children with SCA in this study, including 185 children hospitalized for a vaso-occlusive crisis in whom AKI was assessed and 65 age-matched children in steady state as a reference group of children with SCA (Figure 1). Overall, 243 children had a stored urine sample for NGAL assessment, and 246 had a uNGAL dipstick test. The mean age (SD) of children enrolled in the study was 8.9 (4.0) years, with 107 (42.8%) of study participants female. The reference group was comparable in age and sex to the hospitalized children (Table 1).

Hospitalized children had lower height-for-age, weight-for-age, and body mass index–for–age z scores than the steady-state reference group and were more likely to have malnutrition, severe anemia, and proteinuria and hematuria by dipstick urinalysis (Table 1). Among the hospitalized children, 24 (13.0%) had a history of stroke, and 78 (42.2%) had been hospitalized in the previous 6 months. Hospitalized children presented to the hospital with a median 3-day history of pain, and the most common locations of pain were the lower limbs, abdomen, and chest (Table 1). Analgesia use for children included paracetamol (93.0%), morphine (86.0%), ibuprofen (83.8%), diclofenac (10.3%), codeine (2.2%), and tramadol (1.1%).

The prevalence of AKI in hospitalized children was 36.2% (n = 67/185). Mortality in the study was 3.2%, with 6 of 185 children dying during hospitalization. AKI was associated with an 8.81-fold increased relative risk of death (95% confidence interval [CI], 1.04–74.23). There were no relationships between nephrotoxic medication use (ibuprofen and/or diclofenac) and AKI (P > 0.05 for all).

### Dipstick versus laboratory uNGAL

**Figure 2:**
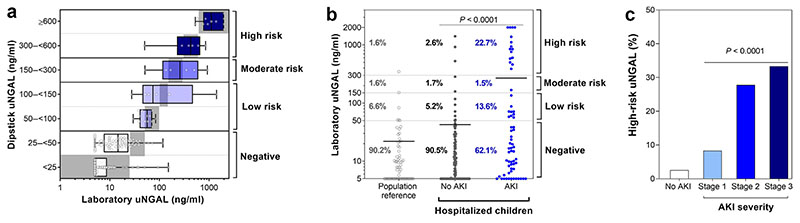
Urine neutrophil gelatinase-associated lipocalin (uNGAL) levels in study population by group and test modality. (**a**) Graph comparing uNGAL concentrations using the semiquantitative dipstick test and the laboratory uNGAL levels. The individual results are depicted by the white circle, with a box plot showing the median (interquartile range) and the whiskers denoting the minimum and maximum values. The dark gray shaded area represents the range for the dipstick test as it relates to the quantitative laboratory values. (**b**) Scatterplot with a bar at the median, depicting uNGAL values measured in the laboratory by enzyme-linked immunosorbent assay (ELISA) in hospitalized children with a vaso-occlusive crisis based on acute kidney injury (AKI) status compared with steady-state outpatient children with sickle cell anemia presenting for routine care. The test results were categorized into negative (≤50 ng/ml), low risk (51–149 ng/ml), moderate risk (150–299 ng/ml), and high risk (≥300 ng/ml). Median uNGAL levels were significantly higher in children with AKI compared with children with no AKI (*P* < 0.0001). (**c**) Bar chart presenting the frequency of high-risk uNGAL levels (≥300 ng/ml) by ELISA in children based on AKI severity.

Levels of uNGAL by dipstick were strongly correlated with enzyme-linked immunosorbent assay—based uNGAL levels, with a nonparametric rank correlation of 0.71 in hospitalized children and 0.88 in children with SCA in steady state (Table 2). The uNGAL levels were categorized on the basis of previously established cutoffs^28^:negative(≤50 ng/ml), low risk (51-149 ng/ ml), moderate risk (150–299 ng/ml), and high risk (≥300 ng/ml; Figure 2). Among children with results by both test modalities, 194 of 242 (80.2%) tested negative by both the dipstick and reference uNGAL test, and there was 71.4% (15 of 21) agreement in children categorized as high risk by both test modalities.

### uNGAL levels are elevated in hospitalized children with AKI

uNGAL levels were comparable between hospitalized children and steady-state outpatient children with SCA (Figure 2). The median laboratory-determined uNGAL concentration was 7.9 (IQR, 5.0–18.2) ng/ml in the reference group of children with SCA in steady state compared with 9.8 (IQR, 5.0–33.5) ng/ml in children hospitalized with a vaso-occlusive crisis (*P* = 0.126). The percentage of children with a positive laboratory uNGAL test result (>50 ng/ml) was 9.8% in the reference group compared with 19.8% in hospitalized children (*P* = 0.08).

**Figure 3:**
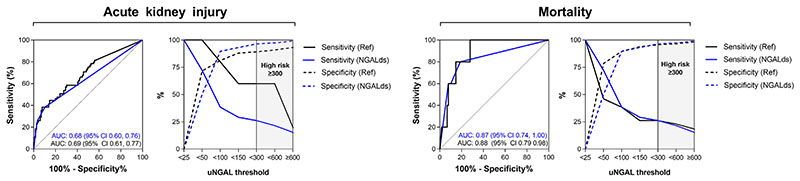
Receiver operating characteristic curves and sensitivity and specificity plots depicting the performance of urine neutrophil gelatinase-associated lipocalin (uNGAL) to diagnose acute kidney injury (AKI) and predict mortality in children with sickle cell anemia hospitalized with a pain crisis. The performance of a laboratory uNGAL test (black), and the point-of-care NGAL dipstick test (blue) is depicted for its ability to diagnose AKI (left) and predict mortality (right) by a receiver operating characteristic curve. In addition, the percentages of sensitivity and specificity of the tests across different test thresholds are depicted in a sensitivity and specificity plot. AUC, area under the curve; CI, confidence interval; NGALds, uNGAL adapted to a dipstick test; Ref, reference.

Consistent with uNGAL as a biomarker of AKI, uNGAL levels were higher in children with AKI compared with children without AKI, with 37.9% of children with AKI having a positive laboratory uNGAL test result (>50 ng/ml) compared with 9.5% in children without AKI (*P* < 0.0001). Furthermore, among children with AKI, 22.7% had a high-risk laboratory NGAL test result (≥300 ng/ml) compared with 2.6% in children without AKI (*P* < 0.0001; Figure 2). The frequency of high-risk uNGAL tests increased across AKI stages, with 8.3% of children with stage 1 AKI, 27.8% of children with stage 2 AKI, and 33.3% of children with stage 3 AKI having a high-risk laboratory uNGAL test (*P* < 0.0001; Figure 2). NGAL had moderate diagnostic accuracy for creatinine-defined AKI, with comparable performance between the reference and dipstick tests (area under the curve [95% CI]: reference, 0.69 [0.61–0.77]; dipstick, 0.68 [0.60–0.76]; Figure 3). Children with a high-risk dipstick uNGAL test had a 2.28-fold increased risk of AKI (95% CI, 1.61–3.23), adjusting for age and sex.

### uNGAL predicts mortality in children hospitalized with a vaso-occlusive crisis

**Figure 4:**
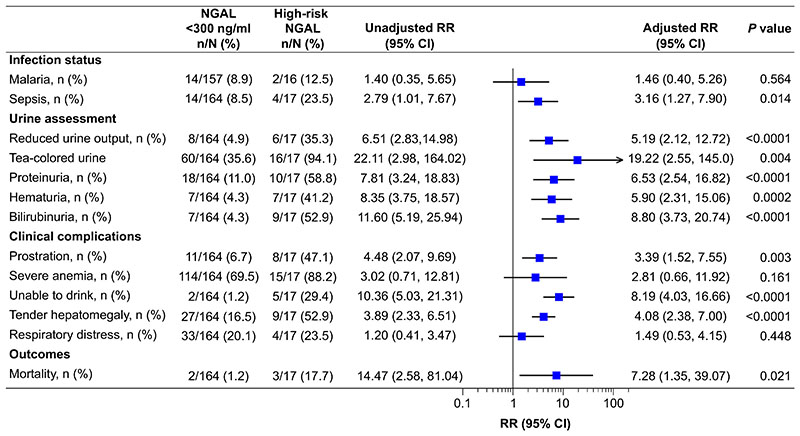
Forest plot depicting the relationship between infections and clinical signs and symptoms of disease severity and a high-risk neutrophil gelatinase-associated lipocalin (NGAL) test. Plot depicting the frequency of high-risk NGAL test results based on infection status, urine assessment, clinical complications, and mortality. The relative risk (RR) is generated from a Poisson regression model with robust variance estimates, with adjusted models including participant age and sex. CI, confidence interval; n, number; N, total number.

We evaluated whether tubular injury was associated with increased mortality over hospitalization (Figures 3 and 4). There was a significant increase in median levels of uNGAL at admission in children who died (median, 263.0 ng/ml [IQR, 26.2–581.8]) compared with survivors (median, 9.6 ng/ml [IQR, 5.0–29.6]; *P* = 0.002). Overall, 60% of children who died had a positive uNGAL dipstick test result. uNGAL had good performance in predicting death, with areas under the curve of >0.85 by both test modalities (area under the curve [95% CI]: reference, 0.85 [0.74–0.95]; dipstick, 0.87 [0.74–0.1.00]; Figure 3). Mortality among hospitalized children was 1.2% (2/164) in children without a high-risk NGAL dipstick test and 17.7% (3/17) in children with a high-risk NGAL dipstick test, corresponding to a relative risk of 7.28 (95% CI, 1.35–39.07), adjusting for participant age and sex (Figure 4).

### Other clinical signs associated with a high-risk NGAL test

We further evaluated the admission findings associated with a high-risk uNGAL dipstick result (Figure 4). Clinical signs and symptoms associated with a high-risk NGAL test included prostration, tender hepatomegaly, a history of being unable to drink, and having reduced urine output, tea-colored urine, or sepsis (Figure 4). Laboratory findings associated with a high-risk NGAL test included proteinuria, hematuria, or bilirubinuria (Figure 4). The presence of malaria, severe anemia, or respiratory distress was not associated with a high-risk NGAL test.

## Discussion

In this study of Ugandan children with SCA, children hospitalized with a vaso-occlusive crisis had comparable uNGAL levels to age-matched children with SCA attending the sickle cell clinic for routine follow-up care. However, among children hospitalized with a vaso-occlusive crisis, uNGAL levels were higher in those with AKI, were increased across AKI stages, and predicted mortality. Certain clinical features in hospitalized children, including sepsis, prostration, tender hepatomegaly, and urine dipstick abnormalities, were associated with high-risk uNGAL. A high-risk NGAL result was associated with a 7.28-fold increased risk of mortality, adjusting for age and sex. More important, the performance of a semiquantitative point-of-care uNGAL test was strongly correlated with continuous uNGAL levels using a laboratory assay and had comparable performance in discriminating between children with AKI and in predicting mortality. The present study suggests that dipstick uNGAL tests may have utility in low- and middle-income countries (LMICs) to identify children with AKI in populations at risk for AKI and mortality.

In the present study, uNGAL levels were strongly correlated between the semiquantitative dipstick test on fresh urine and quantitative levels measured on stored samples. The test modalities had comparable diagnostic accuracy in identifying AKI and predicting mortality. Urine diagnostics have the advantage of being noninvasive. Dipstick tests can be conducted at the bedside and are a preferred test modality in LMICs, where centralized laboratory testing is limited. Widespread use of point-of-care tests in LMIC settings has improved access to diagnostics and has improved clinical management of childhood illnesses (e.g., malaria and HIV).^43^ Advantages of point-of-care tests include ease of use, nonreliance on electricity, temperature stability, acceptability among end users,^44^ and low cost, which makes them ideal for use in resource-limited settings. Urine biomarkers of AKI may facilitate risk stratification or “prognostic enrichment” of children^45^ for early implementation of the KDIGO bundle of care or STOP AKI protocol to prevent AKI progression.^46^

NGAL is a well-established marker of AKI with better performance reported in children compared with adults.^26,27^ In the context of this cohort, the diagnostic accuracy of uNGAL for AKI was consistent with estimates from adult populations, which may reflect preexisting kidney disease in children with SCA. However, using an imperfect reference standard of serum creatinine to diagnose AKI complicates our assessment of uNGAL as a biomarker of AKI. Although there are limitations in creatinine-based diagnosis of AKI that apply to all populations (e.g., delayed increase in creatinine and frequent unknown baseline creatinine), there are additional barriers in LMICs, including a higher prevalence of under-nutrition^38^ that impacts the accuracy of approaches to estimate baseline creatinine. These issues are further exacerbated in children with SCA who may have hyperfiltration, chronic kidney disease, and altered tubular handling of creatinine.^21^ In the present study, 87 (47.0%) of the hospitalized children had a baseline creatinine below the assay’s detection limit and were assigned a value of 0.19 mg/dl. With low creatinine values, imprecision in creatinine measurement can lead to inaccuracies in AKI diagnosis.^47^

Although the number of deaths in this study was limited, the relationship between uNGAL and mortality was strong. These results are consistent with a study of trauma-related AKI in Malawi, where a positive uNGAL test result was strongly predictive of mortality.^28^ Consistency in findings between populations in whom the etiology of AKI is likely to differ supports the generalizability of the results. Although the optimal cutoffs to effectively risk stratify children require validation in other populations and settings, the results suggest uNGAL may be able to identify children with AKI at increased risk of death who may benefit from additional creatinine monitoring. Although point-of-care tests are particularly attractive in LMICs, they can also be leveraged in high-income settings to support rapid clinical decisions. A study of adults presenting to an emergency department in New York City found that the use of uNGAL dipstick tests could rule out AKI.^48^ The use of uNGAL dipsticks was also able to rule out AKI in Malawi, with a specificity of 73.5% and a negative predictive value of 90.2%.^28^ Implementation of uNGAL dipsticks in community settings may improve risk stratification of patients and increase AKI recognition, awareness, and treatment.

In children following cardiac surgery without AKI, uNGAL levels increased within 12 hours of administering nonsteroidal anti-inflammatory drugs, with sustained increases in uNGAL among children receiving multiple nonsteroidal antiinflammatory drug doses.^49^ These data suggest uNGAL may also have clinical utility to identify subclinical AKI associated with structural injury to the kidney in the absence of creatinine changes.^49^ Nonsteroidal anti-inflammatory drugs were administered at least once in 86.5% of hospitalized children in the study, and although it was not associated with the development of AKI using creatinine, serial measures of uNGAL were not available to assess subclinical AKI. In addition, creatinine testing in the study was conducted using a point-of-care test, with the results immediately available to the treating clinician, and this may have impacted nephrotoxic medication use in the study. Among children with SCA enrolled in a clinical trial at the same site, vaso-occlusive crises accounted for 42% of hospitalizations, with children experiencing several vaso-occlusive events per year.^11^ Given the incidence of vaso-occlusive crisis in children with SCA and frequent exposure to nonsteroidal anti-inflammatory drugs, additional monitoring tools are needed to identify subclinical and clinical AKI to guide medical management.

Limitations of this study include a single assessment of uNGAL on admission. Although the sample size was adequate to assess AKI in the study population, it was not powered to assess mortality. As such, we were unable to rigorously evaluate the relationship between a high-risk uNGAL result and mortality, adjusting for potential confounders because of the limited number of deaths. Additional studies are needed to evaluate the impact of hemolysis on uNGAL detection and the performance of uNGAL tests in other populations with intravascular hemolysis (e.g., malaria). The results need to be prospectively validated in other settings and causes of AKI but are consistent with reports of uNGAL-related mortality prediction in Malawian children with trauma-related AKI.^28^ Although the present study showed a relationship between uNGAL levels and severe AKI, additional studies are needed to assess the ability of uNGAL to predict persistent AKI. Strengths of this study included the prospective recruitment of a cohort of children with SCA in sub-Saharan Africa, where most children living with SCA reside. Serial creatinine measures enabled us to define AKI in a population in whom chronic kidney disease prevalence may be high, but baseline creatinine is unknown. Finally, the dipstick uNGAL results were compared with a quantitative enzyme-linked immunosorbent assay and comparable to studies that validated the uNGAL dipstick test against a clinical test.

Overall, this study demonstrates that dipstick uNGAL tests correlate strongly with laboratory reference results, and the dipstick test has comparable diagnostic and prognostic accuracy to the laboratory test. Additional studies are needed to evaluate whether uNGAL dipstick tests can effectively risk stratify children to rule out AKI in low-risk children, identify children in whom preventive measures may improve outcomes (e.g., avoiding nonessential nephrotoxins), and identify high-risk children who may need more frequent monitoring (e.g., repeated uNGAL measures and creatinine monitoring), supportive care, and a nephrology referral. The development of AKI biomarkers that can be used in hospital or outpatient settings in “at-risk” populations has the potential to transform AKI care and outcomes. By focusing efforts to validate low-cost AKI biomarkers, we can prioritize a future in which patients with AKI can be easily identified to improve clinical management and promote equity in care across health care settings globally.

## Figures and Tables

**Table 1:** Description of children with sickle cell anemia enrolled in the study

Demographics	Hospitalized children with a vaso-occlusive crisis (n = 185)			Reference group in steady state (n = 65)
	Combined	No AKI	AKI	
Age, yr	8.9 (5.9 to 11.8)	8.0 (5.1 to 11.3)	10.0 (7.3 to 12.4)	8.8 (5.9 to 12.1)
Age categories, yr				
<5	36 (19.5)	28 (23.7)	8 (11.9)	12 (18.5)
5–10	73 (39.5)	47 (39.8)	26 (38.8)	26 (40.0)
>10	76 (41.1)	43 (36.4)	33 (49.3)	27 (41.5)
Female sex	77 (41.6)	42 (35.6)	35 (52.2)	30 (46.2)
Height-for-age z score	–1.4 (–2.3 to –0.4)	–1.2 (–2.1 to –0.3)	–1.7 (–2.6 to –1.0)	–0.8 (–1.7 to –0.3)
Weight-for-age z score^a^	–1.5 (–2.0 to –0.5)	–1.3 (–2.0 to –0.2)	–1.6 (–2.2 to –0.8)	–0.7 (–1.2 to –0.3)
Weight-for-height z score^a^	–1.6 (–2.2 to –0.4)	–1.6 (–2.2 to –0.3)	–1.6 (–2.6 to –1.3)	0.0 (–0.7 to 0.3)
BMI-for-age z score^a^	–1.3 (–2.3 to –0.4)	–1.3 (–2.0 to –0.4)	–1.2 (–2.7 to –0.2)	–0.7 (–1.2 to –0.1)
MUAC, cm	16.0 (15.0 to 17.8)	16 (14.8 to 17.4)	16.7 (15.2 to 18.2)	16.6 (15.3 to 19.0)
HIV infection	1 (0.5)	0 (0.0)	1 (1.5)	0 (0.0)
**Sickle cell-related complications**				
Splenomegaly	18 (9.7)	9 (7.6)	9 (13.4)	4 (6.2)
Severe anemia	131 (70.8)	77 (62.3)	54 (80.6)	19 (29.7)
Hypertension	34 (18.4)	21 (17.8)	13 (19.4)	9 (13.9)
**Pain assessment**				
Pain score				—
FLACC-R, ≤3yr(n = 18)	4 (4 to 8)	4 (4 to 6)	4 (4 to 8)	
Wong-Baker, >3–7yr(n = 89)	6 (4 to 8)	6 (4 to 8)	6.5 (4 to 8)	
Numeric scale, ≥8yr(n = 78)	6 (4 to 8)	6 (4 to 8)	6 (4 to 8)	
Overall	6 (4 to 8)	6 (4 to 8)	6 (4 to 8)	
Location of pain				—
Chest	64 (34.6)	41 (34.8)	23 (34.3)	
Abdomen	77 (41.6)	50 (42.4)	27 (40.3)	
Back	55 (29.7)	33 (28.0)	22 (32.8)	
Lower limb	120 (64.9)	74 (62.7)	46 (68.7)	
Upper limb	56 (30.3)	34 (28.8)	22 (32.8)	
Other	7 (3.8)	4 (3.4)	3 (4.5)	
Duration of pain, d	3 (2 to 4)	3 (2 to 4)	3 (2 to 4)	—
**Kidney function**				
Dipstick proteinuria	28 (15.1)	9 (7.6)	19 (28.4)	2 (3.1)
Dipstick hematuria	14 (7.6)	4 (3.4)	10 (14.9)	0 (0.0)
Albuminuria^b^				
Microalbuminuria	55 (31.4)	31 (27.9)	24 (37.5)	15 (23.1)
Macroalbuminuria	15 (8.6)	6 (5.4)	9 (14.1)	2 (3.1)
Enrollment creatinine, mg/dl	0.3 (0.19 to 0.4)	0.2 (0.19 to 0.3)	0.19 (0.19 to 0.3)	0.3 (0.2 to 0.4)
Enrollment cystatin C, mg/L	0.8 (0.6 to 1.0)	0.8 (0.6 to 0.9)	1.0 (0.7 to 1.3)	0.9 (0.8 to 1.1)
Enrollment eGFR (creatinine and cystatin C)^c^	133 (104 to 160)	146 (124 to 167)	99 (72 to 133)	118 (100 to 149)
**Outcome**				
Died in hospital	6 (3.2)	1 (0.9)	5 (7.5)	—

AKI, acute kidney injury; BMI, body mass index; eGFR, estimated glomerular filtration rate; FLACC-R, revised face, legs, activity, cry, and consolability; MUAC, mid-upper arm circumference.

a Weight-for-age data available for children aged ≤10 years (n = 150); weight-for-height data available for children aged <5 years (n = 51); and BMI-for-age data available for children aged ≥5 years (n = 202).

b Microalbuminuria was defined as a urine albumin-to-creatinine ratio of 3 to ≤30 mg/mmol, and macroalbuminuria was defined as a urine albumin-to-creatinine ratio >30 mg/mmol.

c eGFR calculated as follows: eGFR = 39.8 * [(height/creatinine)^0.456^] * [(1.8/cystatin C)^0.418^] * [(30/blood urea nitrogen)^0.079^] * (1.076^male^) * [(height/1.4)^0.179^].^39^

Data presented as median (interquartile range) or n (%).

**Table 2:** Categorical comparisons of uNGAL values at enrollment in hospitalized children and a reference group of children in steady state

	Laboratory-based uNGAL assessment in children with sickle cell anemia										
	Hospitalized children (n = 181)						Reference group in steady state (n = 61)				
Risk category	Negative	Low risk	Moderate risk	High risk	Rho		Negative	Low risk	Moderate risk	High risk	Rho
**Dipstick uNGAL**										
Negative	140	4	0	0	0.71		54	1	1	0	0.88
Low risk	5	9	1	1			1	2	0	0	
Moderate risk	0	1	1	2			0	1	0	1	
High risk	0	1	1	15			0	0	0	0	

uNGAL, urine neutrophil gelatinase-associated lipocalin. Negative (≤50 ng/ml), low risk (51–149 ng/ml), moderate risk (150–299 ng/ml), and high risk (≥300 ng/ml).
